# Survival Outcomes and Prognostic Factors for Patients in Early Stage Cervical Cancer: A Multicentric Study in Turkey

**DOI:** 10.3390/diagnostics15212757

**Published:** 2025-10-30

**Authors:** Yesim Ozkaya Ucar, Okan Aytekin, Necim Yalcin, Okan Oktar, Hande Esra Koca Yildirim, Gülsah Tiryaki Guner, Mustafa Gokkaya, Mehmet Unsal, Abdurrahman Alp Tokalioglu, Fatih Celik, Fatih Kilic, Burak Ersak, Günsu Kimyon Cömert, Simge Kirmizigul Kerinc, Dilek Yuksel, Caner Cakir, Cigdem Kilic, Ilker Selcuk, Taner Turan, Tayfun Toptas, Sevgi Koc, Alper Karalok, Isin Ureyen, Derman Basaran, Tolga Tasci

**Affiliations:** 1 Department of Gynecologic Oncology, Ankara Bilkent City Hospital, University of Health Sciences, 06800 Ankara, Turkey; 2 Department of Gynecologic Oncology, Antalya Training and Research Hospital, Faculty of Medicine, University of Health Sciences, 07100 Antalya, Turkey; 3 Department of Gynecologic Oncology, Etlik Zubeyde Hanim Women’s Health Training and Research Hospital, Faculty of Medicine, University of Health Sciences, 06010 Ankara, Turkey; 4 Department of Gynecologic Oncology, Ulus Lıv Hospital, 34340 Istanbul, Turkey; 5 Department of Gynecologic Oncology, Faculty of Medicine, Hacettepe University, 0600 Ankara, Turkey; 6 Department of Gynecologic Oncology, Faculty of Medicine, Bahcesehir University, 34732 Istanbul, Turkey

**Keywords:** cervical cancer, early stage, lymph node metastasis, outcomes, survival

## Abstract

**Background:** To identify prognostic factors related to survival in patients with early-stage cervical cancer treated with radical surgery in six high-volume gynecologic oncology centers in Turkey. **Methods:** This retrospective analysis examined a cohort of 612 patients diagnosed with cervical cancer who underwent type II/III radical hysterectomy and pelvic lymphadenectomy, with or without para-aortic lymphadenectomy at six gynecologic oncology centers. A total of 537 patients between 1993 and 2023 were included. According to the 2009 FIGO staging system, 411 patients (76.5%) were stage IB1, 76 (14.2%) were stage IB2, 40 (4.7%) were stage IIA1, and 10 (1.9%) were stage IIA2. Patients underwent either type II or type III radical hysterectomy with pelvic lymphadenectomy, with para-aortic lymphadenectomy performed in 93.1% of cases. Among the 537 patients, 258 (48%) underwent type II radical hysterectomy and 279 (52%) underwent type III. Univariate and multivariate analyses of 5-year overall survival (OS) and 5-year disease-free survival (DFS) were performed. **Results:** In the entire cohort, 258 (48%) patients underwent radical surgery alone, while 279 (52%) patients underwent radical surgery followed by adjuvant therapy. The 5-year DFS and 5-year OS rates were 85.3% and 98.4%, respectively. In the multivariate logistic analysis, lymph node metastasis was identified as an independent prognostic factor for DFS and OS. **Conclusions:** Lymph node metastasis was the most important prognostic factor for survival in this large multicenter Turkish cohort. These findings highlight the prognostic value of nodal status, stromal invasion, margin status, and LVSI, while underscoring the importance of tailored adjuvant treatment strategies.

## 1. Introduction

Cervical cancer remains a major global health concern and is among the most common cancers in women worldwide, although screening programs have reduced its incidence in developed regions, recurrence and mortality rates remain considerable in global estimates [1]. The most recent GLOBOCAN 2020 report indicates approximately 604,000 new cases and 342,000 deaths worldwide, placing cervical cancer among the leading causes of cancer-related mortality in women [1]. The 5-year survival rate for cervical cancer is 80% when detected in the early stages, specifically International Federation of Gynecology and Obstetrics (FIGO) stage I-II [2]. Taken together, these figures underscore the ongoing global significance of the disease and the need for precise risk stratification.

For early-stage disease, radical hysterectomy with pelvic lymph node dissection or definitive radiotherapy, with or without concurrent chemotherapy, is regarded as the standard treatment [3]. Extensive investigations have been conducted to analyze the impact of clinical and pathological variables on survival outcomes. Reported prognostic factors include lymph node metastases, tumor size, cervical stromal invasion, lympho-vascular space invasion (LVSI), histological subtype, and direct tumor extension to the vagina, parametrium, or surgical border involvement [4,5]. Although not directly included in the current staging system, these clinicopathological factors remain crucial in determining the need for adjuvant therapy after surgery [6].

Cervical cancer is almost entirely caused by persistent infection with oncogenic HPV types, particularly HPV 16 and 18, which account for the vast majority of cases [7]. The implementation of vaccination programs developed against these viral strains has brought about a significant transformation in the field of cervical cancer prevention. Population-based data from Europe and North America have demonstrated a marked decline in high-grade cervical lesions among vaccinated cohorts; however, vaccination coverage remains suboptimal in many low- and middle-income regions, where cervical cancer continues to be a major cause of cancer-related mortality [8,9].

Screening methods such as Pap smear cytology and, more recently, HPV DNA tests have played a critical role in reducing mortality by detecting precancerous lesions before they progress [10]. However, outcomes are not the same for every patient; some patients diagnosed at an apparently early stage still experience recurrence. This highlights the need for additional prognostic indicators beyond just the classic histopathological findings [3]. Beyond traditional factors emerging evidence suggests that molecular features such as p16 overexpression, PD-L1 status, and host immune response profiles may make risk stratification in cervical cancer more sensitive [11,12]. Integrating such biomarkers with classical clinicopathological variables has the potential to refine prognostic assessment and personalize adjuvant treatment decisions.

Surgery remains the mainstay of treatment for early-stage disease, but fertility-sparing interventions such as radical trachelectomy are increasingly being performed in carefully selected young patients with small tumors [13]. In addition, organ-preserving modalities under investigation, such as photodynamic therapy (PDT) have been explored in cervical intraepithelial lesions and microinvasive cases, but have also been proposed as potential investigational options in the context of organ preservation for very early-stage cervical cancer [14]. The role of minimally invasive radical hysterectomy, which was previously widely adopted, was re-evaluated after the LACC trial reported poorer survival compared with open surgery [15]. These results highlighted the prognostic importance of the surgical approach. Overall, current therapeutic options range from definitive surgery or radiotherapy to fertility- and organ-preserving strategies in select patients, reinforcing the importance of accurate baseline risk assignment.

In this study, we aimed to analyze the clinical, surgical, and pathological characteristics of cervical cancer patients with FIGO IB1-IIA2 who underwent radical hysterectomy and lymphadenectomy with/without adjuvant therapy, as well as identify prognostic markers associated with survival outcomes. This investigation represents one of the largest contemporary multicenter cohorts in Turkey, comprising 537 patients from six high-volume gynecologic oncology centers. By integrating surgical, pathological, and survival data, it provides robust, externally valid estimates of disease-free and overall survival and clarifies the prognostic weight of nodal status alongside other clinicopathological variables within a modern treatment framework.

## 2. Materials and Methods

**Figure 1:**
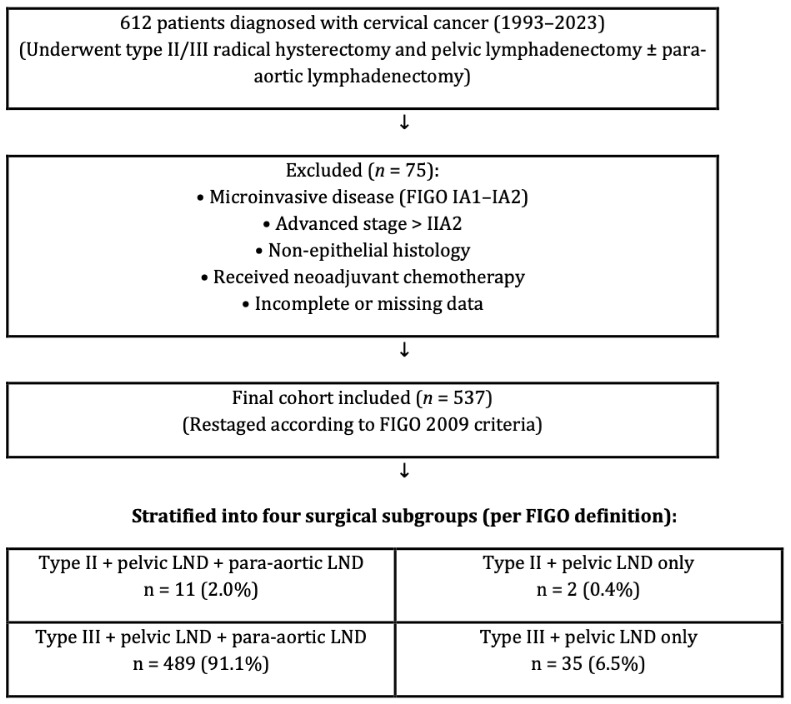
Study flow diagram showing patient inclusion, exclusion, and stratification into four surgical subgroups according to the FIGO classification of radical hysterectomy (Type II or III) and the extent of lymphadenectomy (pelvic ± para-aortic).

This retrospective analysis examined a cohort of 612 patients diagnosed with cervical cancer who underwent type II/III radical hysterectomy and pelvic lymphadenectomy, with or without para-aortic lymphadenectomy. The investigation was conducted at six gynecologic oncology centers between 1993 and 2023. All surgeries were performed by gynecologic oncologists trained at a single institution certification program so homogenization in surgeries could be obtained. Although this represents a relatively long inclusion period, we considered it necessary in order to achieve an adequate sample size from six high-volume institutions. To minimize potential heterogeneity arising from changes in treatment standards, surgical techniques, and diagnostic technologies over time, all patients were restaged uniformly according to the FIGO 2009 classification and pathological evaluation was standardized across centers. A total of 537 patients were eligible after exclusions, contributed from six independent centers. To ensure transparency while maintaining institutional anonymity, the distribution of patients across the centers is provided in Table 1. Patient information was obtained by utilizing the computerized database system, medical files, and pathology reports. Detailed inclusion and exclusion criteria are summarized in Table 2. In brief, eligible patients were women with histologically confirmed cervical carcinoma (FIGO 2009 stages IB1–IIA2) treated with primary radical hysterectomy and pelvic lymphadenectomy. Patients were excluded if they had microinvasive disease, advanced stage beyond IIA2, non-epithelial histology, secondary cervical involvement due to metastasis from other gynecologic malignancies (e.g., endometrial or ovarian carcinoma) synchronous primary tumors, uncertain adjuvant therapy status, receipt of neoadjuvant chemotherapy, preoperative radiotherapy, or incomplete medical records. Importantly, neoadjuvant chemotherapy prior to surgery was an exclusion criterion, and therefore no patients receiving such treatment were included in the final analysis. For clarity, “surgery” in this study refers to type II or type III radical hysterectomy with pelvic lymphadenectomy, with or without para-aortic lymphadenectomy, as illustrated in Figure 1. “Synchronous primary tumors” were defined as histologically distinct gynecologic malignancies diagnosed concurrently with cervical carcinoma. A study flow diagram summarizing patient inclusion, exclusion, and stratification into surgical subgroups (type II/III radical hysterectomy with or without para-aortic lymphadenectomy) is presented in Figure 1. The local institutional ethical board evaluated and approved the research procedure (approval: E2-23-3801/12.04.2023).

Gynecological oncologists performed all surgical procedures. The extent of surgery was determined according to patient- and disease-related factors. Type II or type III radical hysterectomy was performed via open laparotomy or, in selected cases, minimally invasive approaches. Pelvic lymphadenectomy was systematically performed, and para-aortic lymphadenectomy was added at the discretion of the senior surgeon, typically up to the level of the inferior mesenteric artery or left renal vein. In patients with significant comorbidities or unfavorable intraoperative conditions, lymphadenectomy was limited to the pelvic region. Bilateral salpingo-oophorectomy was performed based on patient age, ovarian status, and surgeon preference.

Cervical tumors were classified as; squamous cell carcinoma (*keratinizing*, *non-keratinizing*, *papillary*, *warty*, *verrucous*, *basaloid*, *lymphoepithelial*), adenocarcinoma (*endocervical*, *mucinous*, *endometrioid*, *clear cell*, *serous*, *mesonephric*, *villoglandular*) and other (*adenosquamous*, *undifferentiated*, *adenoid basal*, *adenoid cystic*) types. Histopathological evaluation was carried out according to the 2020 World Health Organization (WHO) criteria [16]. The tumor size was determined by the tumor’s greatest diameter after fixation in paraffin blocks. Deep stromal invasion was characterized by a tumor invading the exterior half of the cervical stroma (>50% of full thickness). In hematoxylin and eosin stained pathologic sections, LVSI was described as tumor cells or cell clusters adhering to vascular walls containing both tumor and healthy tissue in the surrounding area. Surgical border involvement was defined as tumor positivity within a 5 mm margin of the pathological specimen, whereas vaginal metastasis was defined as the presence of the tumor anywhere in the vagina. The patients were staged according to the FIGO 2009 criteria [17].

Baseline demographic and clinical characteristics (age, FIGO stage, histological subtype, tumor size, depth of invasion, LVSI, margin status, etc.) were compiled into a comprehensive table (Table 3) to facilitate comparison with other studies.

Additional subgroup distribution according to the extent of radical hysterectomy and lymphadenectomy is summarized below (Table 4).

Recurrence distal to the pelvic inlet was considered pelvic recurrence, whereas recurrence between the pelvic inlet and diaphragm was considered upper-abdominal recurrence. The remaining failure categories were classified as extra-abdominal recurrences such as recurrences in the liver parenchyma, skin, and bone. Among the 60 patients with recurrence, 36 cases occurred outside the pelvic cavity and were categorized as extra-abdominal recurrence.

Each center’s tumor board made judgments on adjuvant treatment. Adjuvant therapy was defined strictly as treatment administered after primary surgery; patients who had received neoadjuvant chemotherapy were not included in this category. Following surgery ± adjuvant therapy, all patients were entered into a routine surveillance program, with visits scheduled every three months for the first two years, every six months for the next three years, and every year thereafter. On the basis of their symptoms, patients with a suspicion of disease recurrence underwent additional radiological evaluation. Disease-free survival (DFS) was defined as the time between the initial surgery and recurrence, or the time between the initial surgery and the last follow-up visit in the absence of recurrence. Overall survival (OS) was defined as the length of time between initial surgery and mortality due to the disease or the last follow-up visit.

### Statistical Analysis

All statistical analyses were performed using SPSS version 22.0 (IBM SPSS Inc., Chicago, IL, USA). Continuous variables were expressed as mean ± standard deviation or median (range), and categorical variables as number (percentage). Survival estimates for DFS and OS were calculated with the Kaplan–Meier method, and differences were compared with the log-rank test [18]. Variables significant in univariate analyses, or deemed clinically important, were entered into a Cox proportional hazards regression model to identify independent prognostic factors, with hazard ratios (HRs) and 95% confidence intervals (CIs) reported [19]. A *p*-value < 0.05 was considered statistically significant. In addition to whole-cohort analyses, patients were stratified into four surgical subgroups (type II ± para-aortic, type III ± para-aortic) for exploratory survival comparisons.

## 3. Results

### 3.1. Patient’s Characteristics

Baseline demographic and clinicopathological characteristics are presented in the Section 2 (Table 3).

### 3.2. Adjuvant Therapy and Survival

In the entire cohort, 258 (48%) patients underwent radical surgery alone, while 279 (52%) patients underwent radical surgery followed by adjuvant therapy. Adjuvant treatment consisted of concomitant chemoradiotherapy (CCRT; n: 146, 27.2%), radiotherapy alone (RT; n: 125, 23.3%), and chemotherapy (CT) followed by RT (n: 3, 0.6%) (Table 3).

The median follow-up period was 36 months (range 1–300 months). The 5-year DFS and 5-year OS rates were 85.3% and 98.4%, respectively. Recurrence was detected in 60 (11.2%) patients, and 28 (5.2%) patients died because of the disease. In patients with recurrence, the mean time between surgery and recurrence was 22 months (range: 1–75 months). In 27 (5%) patients, recurrences were limited to the pelvic region, in 2 (0.4%) patients to the upper abdominal region, and in 12 (2.2%) patients to the extra abdominal region only. Hepatic recurrence, pulmonary recurrence, bone recurrence, and brain recurrence were detected in 8 (1.5%) patients, 21 (3.9%) patients, 6 (1.1%) patients, and 1 (0.2%) patient, respectively. Data on adjuvant treatment and recurrence patterns are shown in Table 5.

### 3.3. Disease-Free Survival Analysis

**Figure 2:**
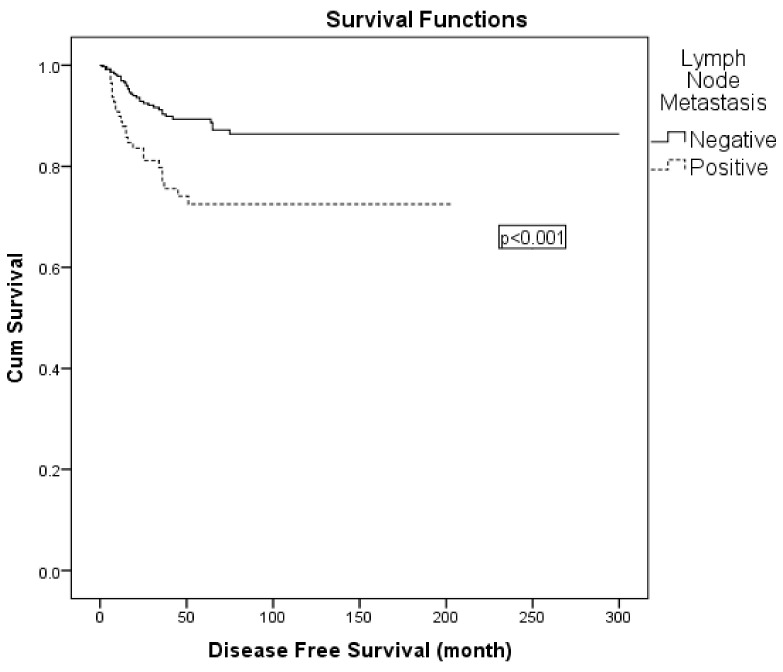
Kaplan–Meier curve for disease-free survival according to lymph node status.

Variables with statistical or clinical relevance were subsequently entered into a multivariable Cox regression model. Given the strong association between nodal positivity and receipt of adjuvant radiotherapy, the latter was excluded from the multivariable model to avoid collinearity. The final model included FIGO 2009 stage (IIA vs. IB), lymph node metastasis (positive vs. negative), surgical margin status (positive vs. negative), and depth of stromal invasion (>50% vs. ≤50%). In this analysis, lymph node metastasis was identified as an independent prognostic factor for DFS (hazard ratio: 2.049; 95% CI: 1.192–3.522; *p* = 0.009) (Table 6; Figure 2). When patients were stratified into four surgical subgroups (type II plus pelvic LND with para-aortic, type II plus pelvic LND without para-aortic, type III plus pelvic LND with para-aortic, and type III plus pelvic LND without para-aortic lymphadenectomy), no statistically significant differences in 5-year DFS were observed between the groups (Table 6). In the comparison of surgical subgroups, adjuvant radiotherapy was administered in 52.8% of all patients, most frequently in those who underwent type III radical hysterectomy with para-aortic lymphadenectomy. However, the difference in adjuvant RT rates between surgical types was not statistically significant (χ^2^ = 4.978, *p* = 0.173).

A subgroup analysis was performed on 127 patients with lymph node metastases. The 5-year DFS rates for negative and positive metastatic paraaortic lymph nodes were 76% and 56%, respectively (*p* = 0.223). Analyzing the relationship between the number of metastatic lymph nodes and survival in this group, patients with single lymph node metastases had a 5-year DFS of 84%, while those with two or more lymph node metastases had a DFS of 63%. Although there was a tendency for a difference, there was no statistically significant difference between the groups (*p* = 0.061).

### 3.4. Overall Survival Analysis

**Figure 3:**
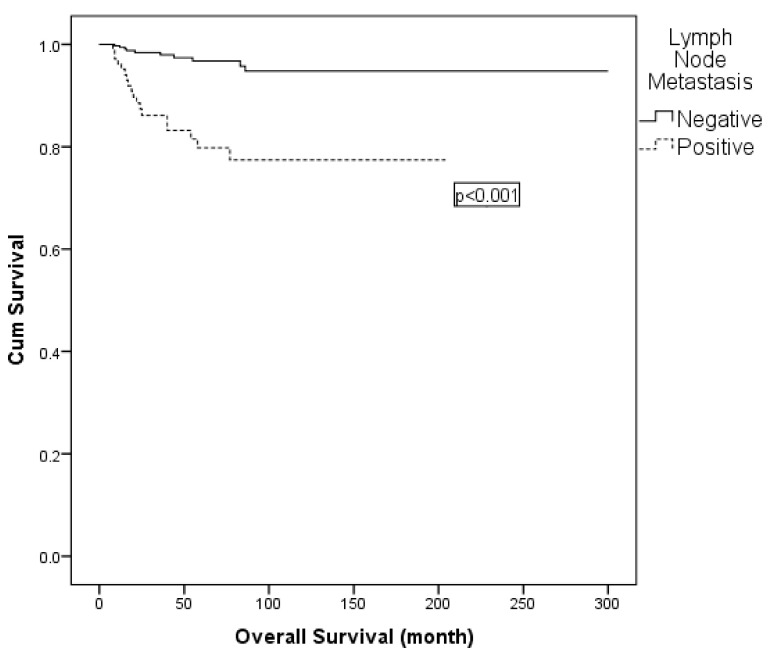
Kaplan–Meier curve for overall survival according to lymph node status.

On univariate analysis, lymph node metastasis, LVSI and adjuvant radiotherapy were found to be effective for OS. In multivariable Cox regression, adjuvant radiotherapy was excluded due to collinearity with nodal status. The final model comprised lymph node metastasis (positive vs. negative) and LVSI (positive vs. negative). Lymph node metastasis remained an independent prognostic factor for OS (hazard ratio: 4.529; 95% CI: 1.974–10.394; *p* < 0.001) (Table 7; Figure 3). Similarly, subgroup comparisons according to surgical type (type II vs. type III, with or without para-aortic lymphadenectomy) did not reveal significant differences in 5-year OS (Table 7).

A subgroup analysis was performed on 127 patients with lymph node metastasis. The 5-year OS rates for metastatic paraaortic lymph nodes that were negative and positive were 80% and 74%, respectively (*p* = 0.383). Analyzing the relationship between the number of metastatic lymph nodes and survival in this group, patients with single lymph node metastases had a 5-year OS of 89%, while patients with two or more lymph node metastases had a 5-year OS of 73%. Although there appeared to be a distinction between the groups, this was not statistically significant (*p* = 0.079).

## 4. Discussion

In this study, we analyzed the clinical, surgical, and pathological characteristics of 537 patients with stage IB1-IIA2 cervical cancer who underwent radical hysterectomy and lymphadenectomy +/− adjuvant therapy. Additionally, the study sought to investigate the potential association between survival outcomes and these aforementioned variables.

Our results show the 5-year DFS and OS rates to be 85.3% and 98.4%, respectively. Recurrence was observed in 11.2% of the patients and 5.2% died as a result of the disease. Lymph node metastasis was found to be an independent prognostic factor for DFS and OS.

Many studies have confirmed a significant correlation between lymph node metastasis and cervical cancer patient prognosis [20,21,22]. Our study revealed that the 5-year DFS and OS for patients with lymph node metastasis were 73% and 80%, compared to 89% and 97% for patients without lymph node metastasis, respectively. In a study of 197 patients with FIGO stage IB-II cervical cancer presented by Ho et al., 5-year DFS was found to be 67.2% in patients with lymph node metastasis whereas individuals without lymph node metastasis exhibited a higher DFS rate of 87.3%. Similarly to our report, lymph node metastasis was determined to be an independent prognostic factor for DFS in that study [23]. In a multivariate analysis of 102 patients with stage IA2-IIB cervical cancer, Obrzut et al. also reported lymph node metastasis as an independent and significant variable for DFS and OS [24].

Recent analyses have also highlighted that the number of positive lymph nodes has an impact on prognosis and that survival loss is associated with nodal burden [25]. Additionally, some studies have reported that the anatomic location of lymph node metastasis (pelvic vs. paraaortic) is prognostically important, but our results did not show a significant difference. This difference may be due to differences in surgical extent and adjuvant treatment approaches between centers [26]. Although some studies have suggested that the extent of surgery (type II vs. type III radical hysterectomy, with or without para-aortic lymphadenectomy) may influence survival outcomes, we were unable to demonstrate such an association in our cohort. This may be related to heterogeneity in surgical practice across centers and time periods, as well as incomplete documentation of surgical details in earlier cases [27]. In our updated subgroup analysis (Table 6 and Table 7), no statistically significant differences were observed between pelvic-only and pelvic + para-aortic lymph-node dissection groups in terms of 5-year DFS (87% vs. 85%, *p* = 0.506) and OS (100% vs. 92%, *p* = 0.232). The slightly lower OS in the type III + PALND subgroup likely reflects that patients with poorer prognostic features such as LVSI, deep stromal invasion, or larger tumor size were more frequently selected for para-aortic dissection. This finding suggests that the extent of lymph-node dissection itself does not independently affect prognosis once baseline risk factors are considered. In the present study, 127 (23.6%) patients had metastatic lymph nodes and the median number of metastatic lymph nodes was 2 (range, 1–37). Our study showed that the 5-year DFS and OS were 84% and 89% for those with only one positive node and 63% and 73% for those with two or more positive nodes, respectively. In a study presented by Ho et al., the 5-year DFS was 60.4% for those with a single positive lymph node, 58.6% for those with two or three positive nodes, 45.9% for those with four or more positive nodes, and 80.4% for those without any positive nodes [23]. Unlike our study, in this study, a statistically significant correlation was found between the number of metastatic lymph nodes and survival. In a study of 2222 patients with FIGO 2009 stage IA-IIB cervical cancer, Zhou et al. reported that patients with 1–2 positive lymph nodes had better OS and DFS outcomes than patients with > 2 positive lymph nodes [28].

Other pathological parameters, such as lymphovascular space invasion (LVSI), depth of stromal invasion, and positive surgical margins, also remain important determinants of recurrence risk. Several studies have shown that LVSI is strongly associated with lymph node metastasis and poorer survival, thus serving as a potential marker of tumor aggressiveness [29,30]. Similarly, tumor size > 4 cm has consistently been reported as a factor increasing the risk of recurrence, as reflected in adjuvant treatment guidelines such as ESGO and NCCN [31]. Furthermore, the ability to achieve complete tumor removal at the initial surgery is a recognized prognostic factor in cervical cancer. However, as R0/R1 status was not systematically recorded in all centers, its impact could not be assessed in this analysis. This is a limitation that warrants consideration in future prospective studies [32]. Recent meta-analyses have further confirmed the prognostic significance of these parameters, particularly in early-stage disease [33].

In our cohort, adjuvant radiotherapy was administered to approximately half of the patients (52.8%), most commonly among those undergoing type III radical hysterectomy with para-aortic lymphadenectomy. Although adjuvant RT rates varied across surgical subgroups, the difference was not statistically significant (*p* = 0.173). This finding likely reflects the higher prevalence of high-risk pathological features in patients selected for extended lymphadenectomy, rather than differences in institutional treatment preferences.

The findings of our study indicate that there was no correlation between the location of the positive lymph nodes (pelvic vs. paraaortic) and DFS or OS. In contrast to our findings, Takeda et al. analyzed the outcomes of 36 lymph node-positive patients and found a correlation between the location of positive lymph nodes (pelvic node excluding common iliac nodes or common iliac nodes vs. paraaortic nodes) and DFS [34].

In the present study, the 5-year OS rate was considerably higher among patients without LVSI than among women diagnosed with LVSI (97 vs. 89%; *p* = 0.030), according to the univariate analysis. However, the results of the multivariate test showed no significance. While no statistically significant difference was seen, those without LVSI also had a higher 5-year DFS rate with a tendency towards significance. Similarly, Obrzut et al. reported in a study that although DFS and OS were higher in patients without LVSI, it was not an independent and significant variables [24]. An unexpected finding in our series was that patients who did not receive adjuvant radiotherapy had higher 5-year OS rates compared to those who did receive radiotherapy. This should not be considered a detrimental effect of radiotherapy. We believe this result is primarily due to the fact that patients who received radiotherapy had high-risk features such as lymph node involvement, margin positivity, or deep stromal invasion. The poorer initial prognosis of this group explains why their survival rates were lower despite receiving additional treatment.

Finally, although molecular prognostic markers such as p16INK4a and PD-L1 were discussed in the context of recent literature, it should be noted that these biomarkers were not analyzed within the present dataset. Their inclusion serves only to contextualize our findings within emerging molecular prognostic frameworks.

Moreover, emerging evidence suggests that additional histopathological and immune-related factors, such as tumor budding, cell nesting, and tumor-infiltrating lymphocytes, may further refine risk stratification in cervical cancer patients [35]. Similarly, scoping reviews focusing on Asian populations have confirmed that classical prognostic factors like histology, LVSI, and nodal status remain relevant even in diverse clinical contexts [36].

Molecular biomarkers are increasingly being evaluated to complement traditional histopathological risk factors. For example, p16INK4a overexpression has been shown in meta-analyses to be associated with improved disease-free and overall survival, as it may be linked to HPV-associated oncogenesis [12]. PD-L1 expression and tumor immune microenvironment profiles are also being investigated for predicting survival and response to adjuvant or immunotherapies [37]. Recent prognostic nomograms designed for younger cervical cancer populations also highlight the role of integrating molecular and clinical factors into predictive models [38]. Integrating these biomarkers into clinical models may increase prognostic accuracy and contribute to the individualization of adjuvant treatment decisions.

Importantly, this multicentric design allows validation of established prognostic factors under diverse real-world surgical and institutional conditions, bridging the gap between controlled single-center studies and everyday clinical practice. By including data from six tertiary gynecologic oncology centers, the study enhances the external validity and generalizability of its findings across heterogeneous treatment settings.

The primary limitation of this study is its retrospective design. The study’s strengths are its multicentric design and large patient population. Secondly, all surgical procedures were conducted by gynecologic oncologists, and then pathologic assessments were evaluated by expert gyneco-pathologists. Compared with more recent multicenter series, our findings remain consistent in demonstrating the prognostic importance of nodal involvement and LVSI, while also underlining the challenges of treatment heterogeneity across institutions [33,38].

Another limitation of the study is the heterogeneity in adjuvant treatment strategies across centers and over time. While this reflects real-world practice, it may account for some variability in the results. However, the multicenter nature of the cohort and long-term follow-up strengthen the external validity of our findings. In addition, detailed data regarding the specific surgical methods (type II vs. type III radical hysterectomy with or without para-aortic lymphadenectomy) were not uniformly available across all centers and time periods. All radical hysterectomies were performed by certified gynecologic oncologists in accordance with FIGO-based standards (Type II or III radical hysterectomy with systematic pelvic lymphadenectomy ± para-aortic dissection). Although R0/R1 status was not uniformly documented in earlier years, all included patients met the criteria for macroscopically complete resection at the time of surgery. For this reason, these parameters could not be included in the multivariate analysis, and we acknowledge this as a limitation. Similarly, information on the completeness of tumor resection (R0 vs. R1 status) was not consistently documented, although it is recognized as an important prognostic factor. Future prospective multicenter studies that include molecular biomarkers and standardize adjuvant treatment protocols will help confirm these observations.

## 5. Conclusions

In summary, our study confirms that lymph node metastasis remains an independent prognostic factor for DFS and OS in patients with early-stage cervical cancer. Although additional clinicopathological variables such as tumor size, depth of invasion, LVSI, and surgical margin status also influence outcomes, their independent predictive value is limited in multivariate models. These findings underscore the importance of accounting for potential collinearity between nodal status and adjuvant treatment variables when interpreting prognostic analyses. Our findings, consistent with recent multicenter studies, reinforce the continued importance of careful surgical staging and comprehensive risk assessment in guiding adjuvant treatment decisions.

## Figures and Tables

**Table 1:** Distribution of patients across participating centers.

Center	Number of Patients (*n*)	Percentage (%)
Center 1	424	79.0
Center 2	34	6.3
Center 3	47	8.7
Center 4	25	4.7
Center 5	2	0.4
Center 6	5	0.9
**Total**	537	100.0

**Table 2:** Inclusion and exclusion criteria for patient selection.

Inclusion Criteria	Exclusion Criteria
Histologically confirmed cervical carcinoma (squamous cell carcinoma, adenocarcinoma, or adenosquamous carcinoma)	Microinvasive cervical cancer (FIGO stage IA1–IA2)
FIGO 2009 stages IB1–IIA2	Advanced stage disease (>IIA2)
Primary treatment with type II or type III radical hysterectomy and pelvic lymphadenectomy (± para-aortic lymphadenectomy)	Non-epithelial cervical malignancies (e.g., sarcoma, lymphoma)
Availability of complete clinicopathological and follow-up data	Synchronous primary tumors
Surgery performed between 1993 and 2023 at one of the six participating centers	Receipt of neoadjuvant chemotherapy prior to surgery
Age ≥ 18 years	Missing or uncertain adjuvant therapy status
Approval by institutional ethics committee and informed consent (where available)	Incomplete clinical, surgical, or pathological records

(a) Secondary cervical carcinoma refers to cervical involvement due to metastasis from other gynecologic malignancies (e.g., endometrial or ovarian carcinoma). (b) Surgery refers to type II or type III radical hysterectomy with pelvic lymphadenectomy, with or without para-aortic lymphadenectomy, as illustrated in Figure 1. (c) Synchronous primary tumors were defined as histologically distinct gynecologic malignancies diagnosed concurrently with cervical carcinoma. (d) Patients who received neoadjuvant chemotherapy or preoperative radiotherapy were excluded.

**Table 3:** Characteristics of entire cohort.

Features		Mean ± SD	Median (Range)
Age		51.7 ± 10.39	(26–79)
Tumor size (mm)		30.6 ± 13.38	(6–80)
Number of removed lymph node		47.5 ± 21.15	(10–128)
Number of metastatic lymph node		3.8 ± 5.27	(1–37)
		***n***	**%**
2009 FIGO stage	IB1	411	76.5
	IB2	76	14.2
	IIA1	40	4.7
	IIA2	10	1.9
Tumor size according to 2018 FIGO stage	≤20 mm	160	29.8
	>20 mm–≤40 mm	291	54.2
	>40 mm	86	16
Tumor type ^1^	Squamous cell carcinoma	409	76.2
	Adenocarcinoma	91	16.9
	Mixed type (squamous cell carcinomaand adenocarcinoma)	6	1.1
	Others (Adenosquamos/Glassy cell) ^2^	31(27/4)	5.8
Parametrial involvement	Negative	452	84.2
	Positive	84	15.6
	Not reported	1	0.2
Surgical border involvement	Negative	499	92.9
	Positive	38	7.1
Lymphovascular space invasion	Negative	221	41.2
	Positive	290	54
	Not reported	26	4.8
Depth of cervical stromal invasion	≤50%	179	33.3
	>50%	340	63.3
	Not reported	18	3.4
Lymph node metastasis	Negative	410	76.4
	Positive	127	23.6
Site of metastatic lymph node	Only pelvic	106	19.7
	Only paraaortic	2	0.4
	Pelvic and paraaortic	19	3.5
Ovarian transposition	Not performed	443	82.5
	Performed ^3^	94	17.5
Ovarian metastasis ^4^	Negative	446	98.2
	Positive	8	1.8

SD: Standard Deviation. ^1^: Tumor type: Defined according to 2020 WHO criteria; ^2^: Others: Adenosquamous cancer, glassy cell cancer; ^3^: n = 94 (82 patients had bilateral ovary transposed to pelvic side wall + 12 patients had unilateral salpingo-oophorectomy and the other ovary transposed to pelvic side wall); ^4^: n = 454 patients (442 patients underwent bilateral salpingo-oophorectomy + 12 patients underwent unilateral salpingo-oophorectomy).

**Table 4:** Distribution of patients according to surgical type and para-aortic lymphadenectomy status.

Surgical Type	*n*	%
Type II plus pelvic LND with para-aortic LND	11	2
Type II plus pelvic LND without para-aortic LND	2	0.4
Type III plus pelvic LND with para-aortic LND	489	91.1
Type III plus pelvic LND without para-aortic LND	35	6.5
**Total**	537	100

**Table 5:** Adjuvant Treatment, Recurrence and Recurrence Pattern.

Parametre		*n*	%
Adjuvant treatment	Not received	258	48
	Received	279	52
Type of adjuvant treatment	CCRT	146	27.2
	Only RT	125	23.3
	CT followed by RT	3	0.6
	Not reported	5	0.9
Recurrence	Negative	477	88.8
	Positive	60	11.2
Recurrence pattern	Only pelvic	27	5
	Only upper abdominal	2	0.4
	Only extra abdominal	12	2.2
	Pelvik + upper abdominal	2	0.4
	Pelvik + extra abdominal	4	0.7
	Upper abdominal + extra abdominal	5	0.9
	Pelvic + upper abdominal + extra abdominal	8	1.5

**CCRT:** Concomitant chemoradiotherapy; **RT:** Radiotherapy; **CT:** Chemotherapy.

**Table 6:** Factors related with disease-free survival.

Univariate Analysis				Multivariate Analysis		
Parametre		5-Year Disease-Free Survival		Recurrence		
		%	*p* Value	Odds Ratio	95% CI	*p* Value
Age ^1^	≤51 years	86	0.779			
	>51 years	85				
Histopathology ^2^	Squamous cell	85	0.904			
	Other ^3^	86				
FIGO 2009 Stage	IB1	88	0.054			
	IB2	83				
	IIA1	70				
	IIA2	67				
FIGO 2009 Stage	IB	87	**0.020**	1 (Reference)	0.739–3.214	0.249
	IIA	70		1.541		
Tumor size ^1^	≤30 mm	87	0.071			
	>30 mm	83				
Lymph node metastasis	Negative	89	**<0.001**	1 (Reference)	1.192–3.522	**0.009**
	Positive	73		2.049		
Number of removed total lymph nodes ^1^	≤44	84	0.273			
	>44	87				
Parametrial involvement	Negative	86	0.949			
	Positive	83				
Surgical border involvement	Negative	87	**0.011**	1 (Reference)	0.604–3.110	0.451
	Positive	64		1.371		
LVSI	Negative	89	0.064			
	Positive	83				
Depth of cervical stromal invasion	≤50%	92	**0.021**	1 (Reference)	0.857–3.157	0.135
	>50%	81		1.645		
Bilateral salpingo-oophorectomy	Not performed	88	0.630			
	Performed	85				
Surgery type	Type II plus pelvic LND without PA LND	50	0.260			
	Type II plus pelvic LND with PA LND	100				
	Type III plus pelvic LND without PA LND	92				
	Type III plus pelvic LND with PA LND	85				
Type of LND	Only pelvic	87	0.506			
	Pelvic and PA	85				
Adjuvant radiotherapy	Not received	90	**0.036**			
	Received	82				
Type of adjuvant radiotherapy ^4^	Only RT	84	0.994			
	CCRT	83				

^1^: Median Value; ^2^: Six patients with mixed-type cancer were excluded; ^3^: Other: Non-squamous cell cancer; ^4^: n = 271 patients (125 patients received only RT versus 146 patients received CCRT); **CI:** Confidence Interval; **CCRT:** Concomitant chemoradiotherapy; **RT:** Radiotherapy; **LVSI:** Lymphovascular space invasion; **LND:** Lymphadenectomy.

**Table 7:** Factors related to overall survival.

Univariate Analysis				Multivariate Analysis		
**Parametre**		5-Year Overall Survival		Death Because of Disease		
		%	*p* Value	Odds Ratio	95% CI	*p* Value
Age ^1^	≤51 years	93	0.928			
	>51 years	92				
Histopathology ^2^	Squamous cell	92	0.471			
	Other ^3^	96				
FIGO 2009 Stage	IB1	93	0.648			
	IB2	94				
	IIA1	89				
	IIA2	75				
FIGO 2009 Stage	IB	93	0.486			
	IIA	87				
Tumor size ^1^	≤30 mm	94	0.162			
	>30 mm	91				
Lymph node metastasis	Negative	97	**<0.001**	1 (Reference)	1.974–10.394	**<0.001**
	Positive	80		4.529		
Number of removed total lymph nodes ^1^	≤44	91	0.306			
	>44	94				
Parametrial involvement	Negative	93	0.456			
	Positive	88				
Surgical border involvement	Negative	93	0.471			
	Positive	89				
LVSI	Negative	97	**0.003**	1 (Reference)	0.756–7.354	0.090
	Positive	89		2.358		
Depth of cervical stromal invasion	≤50%	96	0.072			
	>50%	91				
Bilateral salpingo-oophorectomy	Not performed	94	0.925			
	Performed	92				
Surgery type	Type II plus pelvic LND without PA LND	100	0.628			
	Type II plus pelvic LND with PA LND	100				
	Type III plus pelvic LND without PA LND	100				
	Type III plus pelvic LND with PA LND	92				
Type of LND	Only pelvic	100	0.231			
	Pelvic and PA	92				
Adjuvant radiotherapy	Not received	97	**0.002**			
	Received	89				
Type of adjuvant radiotherapy ^4^	Only RT	91	0.631			
	CCRT	88				

^1^: Median Value; ^2^: Six patients with mixed-type cancer were excluded; ^3^: **Other:** Non-squamous cell cancer; ^4^: n = 271 patients (125 patients received only RT versus 146 patients received CCRT); **CI:** Confidence Interval; **CCRT:** Concomitant chemoradiotherapy; **RT:** Radiotherapy; **LVSI:** Lymphovascular space invasion; **LND:** Lymphadenectomy.

## Data Availability

All data generated or analyzed during this study are included in this published article.

## Abbreviations

OS	overall survival
DFS	disease-free survival
FIGO	International Federation of Gynecology and Obstetrics
LVSI	lympho-vascular space invasion
WHO	World Health Organization
CCRT	concomitant chemo-radiotherapy
RT	radiotherapy
CT	chemotherapy
